# Acute exposure to ultraviolet radiation targets proteins involved in collagen fibrillogenesis

**DOI:** 10.3389/fphys.2024.1352161

**Published:** 2024-03-15

**Authors:** Christopher I. Platt, Callum Stewart-McGuinness, Alexander Eckersley, Loren Wilkins, Michael J. Sherratt

**Affiliations:** ^1^ Division of Cell Matrix Biology & Regenerative Medicine, School of Biological Science, Faculty of Biology, Medicine and Health, The University of Manchester, Manchester, United Kingdom; ^2^ Division of Musculoskeletal & Dermatological Sciences, School of Biological Sciences, Faculty of Biology, Medicine and Health, The University of Manchester, Manchester, United Kingdom; ^3^ School of Medical Sciences, The University of Manchester, Manchester, United Kingdom

**Keywords:** skin, extracellular matrix, ultraviolet, collagen fibrillogenesis, mass spectrometry, post-translational modification

## Abstract

**Introduction:** Exposure to chronic, low-dose UV irradiation (UVR) can lead to premature ageing of the skin. Understanding which proteins are affected by acute UVR and photo-dynamically produced reactive oxygen species (ROS) could help to inform strategies to delay photoageing. Conventional biochemical analyses can be used to characterize UVR/ROS-induced damage on a protein-by-protein basis and we have previously shown using SDS-PAGE that collagen I and plasma fibronectin are respectively resistant and susceptible to physiological doses of UVR. The aim of this study was to screen a complex proteome for UVR-affected proteins.

**Methods:** This study employed a sensitive mass spectrometry technique (peptide location fingerprinting: PLF) which can identify structure associated differences following trypsin digestion to characterize the impact of UVR exposure on purified collagen I and tissue fibronectin and to identify UVR-susceptible proteins in an ECM-enriched proteome.

**Results:** Using LC/MS-MS and PLF we show that purified mature type-I collagen is resistant to UVR, whereas purified tissue fibronectin is susceptible. UV irradiation of a human dermal fibroblast-deposited ECM-enriched proteome *in vitro*, followed by LC/MS-MS and PLF analysis revealed two protein cluster groups of UV susceptible proteins involved in i) matrix collagen fibril assembly and ii) protein translation and motor activity. Furthermore, PLF highlighted UV susceptible domains within targeted matrix proteins, suggesting that UV damage of matrix proteins is localized.

**Discussion:** Here we show that PLF can be used to identify protein targets of UVR and that collagen accessory proteins may be key targets in UVR exposed tissues.

## 1 Introduction

Chronically photoaged skin is characterized macroscopically by the presence of wrinkles, coarseness, and loss of elasticity, and microscopically by fragmentation of dermal interstitial collagens, disorganized aggregation of glycosaminoglycans (GAGs), degradation of fibrillin-rich microfibrils and the presence of elastotic material (Bernstein et al., 1996; Watson et al., 1999; Fligiel et al., 2003). These macroscopic changes are mediated, in part, by cellular mechanisms which are induced following UV irradiation. For example, dermal protein oxidation is increased but antioxidant enzymes, such as catalase, are decreased in photoaged skin (Sander et al., 2002; Zucchi et al., 2022). Furthermore, acute ultraviolet (UV) irradiation of skin *in vivo* or fibroblast cultures *in vitro* leads to an increase in dermal protein carbonylation, thus highlighting a direct link between UV exposure and oxidative damage of dermal proteins (Sander et al., 2002). In addition, exposure of skin to ultraviolet radiation (UVR) induces an inflammatory response in epidermal keratinocytes by stimulation of the inflammasome leading to the activation of the pro-inflammatory cytokine IL-1β by caspase-1 cleavage (Hasegawa et al., 2016). In the dermis, UVR exposure upregulates members of the matrix metalloprotease family (MMP) of ECM-degrading proteases, via AP-1 signalling, which can lead to aberrant remodeling of the dermal matrix (Quan et al., 2010; Oh et al., 2020). However, it is also clear that extracellular matrix (ECM) components of the dermis can undergo direct structural modification as a consequence of exposure to physiological doses of UVR in cell-free systems. We have previously shown that ECM biomolecules which are enriched in UV chromophore amino acid residues (for UVA: disulphide-bonded Cysteine ([Cys], Tryptophan [Trp] and Tyrosine [Tyr]), such as fibrillin microfibrils and plasma fibronectin, are damaged by exposure to broadband UVB, solar simulated radiation (SSR) and UVA (Sherratt et al., 2010). In contrast, chromophore-poor proteins such as collagen I and tropoelastin appear resistant to physiological UVR wavelengths. In subsequent studies we have shown that UV irradiation in both oxygen depleted conditions and in the presence of D_2_O directly damages ECM proteins, indicating that this is primarily mediated by the photodynamic production of reactive oxygen species (ROS) (Hibbert et al., 2019).

Whilst the use of biochemical and biophysical characterization methods such as gel electrophoresis and ultrastructural imaging (Sherratt et al., 2010) can identify specific changes in the electrophoretic behavior or morphology of individual ECM components and macro-molecular assemblies, these techniques are poorly suited to screening complex protein mixtures. Furthermore, their sensitivity to UVR-induced structural changes may be limited; for example, atomic force microscopy of collagen VI microfibrils failed to identify any significant changes in ultrastructure following irradiation with SSR (Eckersley et al., 2020) but the latter study demonstrated that the localized peptide yield, detected by liquid chromatography with tandem mass spectrometry (LC-MS/MS) following tryptic digest, was significantly affected along the structure of the alpha 3 chain of collagen VI. Therefore, the apparent resistance of some dermal components, such as collagen VI, to acute UVR exposure and chronic exposure *in vivo* (Watson et al., 2001) may be due to methodological limitations in sensitivity.

Peptide location fingerprinting (PLF) is a proteomic technique which exploits the variable sensitivity of trypsin-mediated protein cleavage within local regions of higher order protein structure. By quantifying and statistically comparing differences in protein regional peptide abundance between samples, as detected by LC-MS/MS following trypsin digestion, PLF can identify structural heterogeneity as a consequence of, for example, UVR-induced remodeling of fibrillin microfibrils (Sherratt et al., 2010). We have previously shown that the characteristic peptide location fingerprint of many ECM proteins is highly conserved across tissues and species making such fingerprints ideal markers of structural remodeling as a consequence of ageing and/or disease (Ozols et al., 2021b). However, we have not yet employed PLF to screen complex protein mixtures subjected to *in vitro* degradative mechanisms for structurally heterogeneous proteins.

Given the complexity of the dermal matrisome and the heterogeneity in amino acid composition exhibited by ECM proteins (Sherratt et al., 2010) it is unlikely that acellular, UVR-induced protein remodeling is confined to the small number of purified UV-chromophore rich proteins (fibrillin microfibrils, plasma fibronectin and collagen VI alpha 3) studied to date. The aims of this study were to use the sensitivity of PLF to: i) determine if the structure of purified collagen I was resistant UVR exposure, ii) confirm if tissue (as opposed to plasma) fibronectin is UVR-labile and if so to localize these structural changes and iii) identify potential candidates of acellular UVR-mediated degradation in a complex ECM-enriched proteome.

## 2 Materials and methods

### 2.1 Cell culture

Human dermal fibroblast (HDF)-deposited ECM was generated as previously described (Ozols et al., 2021a). HDFs (passage 4) isolated from a biopsy taken from the sun-protected buttock skin of a 23-year-old Caucasian female. Subjects gave their informed consent for inclusion before they participated in the study. The study was conducted in accordance with the Declaration of Helsinki, and the protocol was approved by the University of Manchester Ethics Committee (Ref: 2020-8895-15556). HDFs were cultured in 35 mm diameter plates for 9 days post-confluence in Dulbecco’s modified Eagle’s medium (DMEM) supplemented with a final concentration of 10% (v/v) foetal bovine serum (Gibco), penicillin (100 U/mL) and streptomycin (0.1 mg/mL), GlutaMAX (2 mM), and ascorbate-2-phopshate (50 μg/mL). Medium was replaced every 2–3 days. To remove cells from the deposited ECM, monolayers were washed three times with 0.5 mL phosphate buffered saline (PBS) and incubated with 0.5 mL cell extraction buffer (0.5% v/v TRITON-X 100, 20 mM ammonium hydroxide solution in PBS). Cell removal was judged by light microscopy (Franco-Barraza et al., 2016). Remaining cell debris was removed from cell-depleted ECM by washing three times in PBS. The Final PBS wash was aspirated and the matrices were stored at −80°C until irradiation. To confirm cell removal, ECM was fixed with 100% methanol for 10 min at 4°C, followed by staining with haematoxylin for 5 min at room temp. Images were captured with a camera (Leica MC170-HD) attached to a light microscope (Leica DMi1).

### 2.2 UV irradiation

Protein suspensions were irradiated with broadband UVB of 50 or 500 mJ/cm^2^, using a Philips TL-12 source (Philips) which emits UVC 0.4%, UVB 55.3% and UVA 44.3%. Irradiation with 50 mJ/cm^2^ broadband UVB is equivalent to approximately one minimal erythemal dose (MED), which is the dose required to cause perceptible skin reddening in Fitzpatrick phototype I-III skin (Andreassi et al., 1987). UV exposure times were calculated from irradiances measured using a UVX radiometer (UVX-31 detector – UVR products; Upland, CA, USA) as previously described (Hibbert et al., 2019). Native human type-I collagen from placenta (AbCam) was neutralized by dialysis against 50 mM Tris-HCl/200 mM NaCl (pH 7.5) at 4°C for 4 h with one change of buffer. 4 μL dialyzed type-I collagen (1 mg/mL) or 6 μL human tissue fibronectin (1 mg/mL), isolated from infant foreskin fibroblasts *in vitro* (Merck), were irradiated in the upturned lid of a 0.5 mL polyethylene tube. Control, non-irradiated, protein suspensions (0 mJ/cm^2^) were placed beneath a 3 mm Perspex sheet (SimplyPlastics, Billericay) throughout the irradiation procedure to block UVR. The ability of the Perspex to block UVR was confirmed by placing the UVX radiometer detector beneath the Perspex sheet. For irradiation of HDF-deposited ECM, cell-depleted matrices were covered with 1 mL PBS to prevent dehydration and the ECM irradiated at 100 mJ/cm^2^.

### 2.3 Sodium dodecyl sulfate—polyacrylamide gel electrophoresis (SDS-PAGE)

UV-irradiated protein suspensions were diluted to 0.1 mg/mL in buffer (50 mM Tris, 10 mM CaCl_2_, 150 mM NaCl, 0.05% (w/v) Brij-35, pH 7.5). 200 ng protein was reduced at 85 °C for 10 min and then electrophoresed at 120 V for 2 h (type-I collagen) or 3 h (fibronectin) in a 4%–12% polyacrylamide gel (ThermoFisher), in NuPAGE™ MES SDS running buffer (ThermoFisher). The molecular weight of the sample was estimated by running 2 μL pre-stained molecular weight marker (New England Biolabs) in an adjacent lane. Protein bands were highlighted using silver stain (Pierce™), according to manufacturer’s instructions. Digital images of SDS-PAGE gels were uploaded into ImageJ and relative protein abundance and electrophoretic mobility were quantified by measuring relative band intensity and calculating the average for each experimental group.

### 2.4 Western blotting

Following electrophoresis, protein was transferred onto a 0.45 μm polyvinylidene fluoride (PVDF) membrane (ThermoFisher) at 20 V for 1 h. The PVDF membrane was incubated in blocking buffer: 5% (w/v) non-fat milk in Tris-buffered saline (50 mM Tris/139 mM NaCl (pH 7.6) containing 0.1% (v/v) Tween-20) for 2h with agitation, prior to incubating in blocking buffer containing 1:20,000 dilution of a rabbit anti-fibronectin polyclonal antibody (Proteintech) for 16 h at 4°C with agitation. Following 3X washes in Tris-buffered saline, membrane was incubated in blocking buffer containing 1:20,000 dilution of goat anti-rabbit IgG, HRP-conjugate (Bio Rad) for 2 h at room temperature with agitation. Following 3X washes in Tris-buffered saline, specific antibody binding was detected with ECL substrate (Perkin Elmer), according to manufacturer’s instructions, and the image transferred onto x-ray film (Amersham) in the dark. To develop bands, film was placed into developer (Scientific Laboratory Supplies) for 3 min, tap water for 3 min, and fixer (Scientific Laboratory Supplies) for 3 min.

### 2.5 Sample preparation for mass spectrometry

UV irradiated and control protein suspensions (1 μg total protein) were dialyzed against ultrapure dH_2_O for 4 h at 4°C to remove buffer components, and the dialysate was freeze-dried for 16 h at −50°C and 0.05 hPa (CoolSafe™, Scanvac). For HDF-deposited ECM, PBS was removed from irradiated matrices which were scraped from the base of the plastic well into 100 μL buffer (50 mM Tris, 10 mM CaCl_2_, 150 mM NaCl, 0.05% (w/v) Brij-35, pH 7.5). Buffer containing 30 μg ECM protein was dialyzed against ultrapure dH_2_O and freeze-dried as stated above.

Freeze-dried protein suspensions were resuspended in 5% sodium dodecyl sulfate (SDS)/50 mM triethylammonium bicarbonate (TEAB), pH 7.5. For HDF-deposited ECM, freeze-dried samples were resuspended in 50 mM TEAB/5% SDS (pH 7.5) and sheared at 4°C using the LE220-Plus Focused Ultrasonicator (Covaris, UK) with the following settings: duration (180 s), peak power (500 W), duty factor (37%), cycles per burst (500), average power (185). The homogenization process resulted in the break-up of visible ECM aggregates leaving a uniform protein suspension. Samples were reduced by adding 5 mM (final concentration) dithiothreitol (DTT; in ultrapure water) and incubating at 60°C for 10min. Samples were alkylated by adding 15 mM (final concentration) of iodacetamide (in ultrapure water) and incubating at room temperature in the dark for 30 min. Reactions were quenched by adding 5 mM (final concentration) of DTT.

Samples were acidified with aqueous phosphoric acid (1.2% final concentration) and diluted in 6 X volume of buffer (90% aqueous methanol containing 100 mM triethylammonium bicarbonate, pH 7.1). Samples derived from protein suspension were added to an S-Trap spin column (ProtiFi) and sample collected onto a protein trapping membrane by centrifugation at 4,000 g for 2 min. Due to the high protein concentration, samples derived from HDF-deposited ECM were added to S-trap columns within a 96-well plate setup; however, suspension and ECM protein samples were processed in an identical manner. Samples were washed X 4 with 90% aqueous methanol containing 100 mM triethylammonium bicarbonate, pH 7.1, prior to digestion with proteomic grade trypsin (Promega) (1:10 ratio of trypsin to protein sample) in 50 mM triethylammonium bicarbonate, at 47°C for 1 h. Peptides were eluted by 3 successive washes in 50 mM triethylammonium bicarbonate, 0.1% aqueous formic acid, and 30% aqueous acetonitrile/0.1% formic acid.

For peptide desalting, peptides were added to washed Oligo R3 resin beads (ThermoFisher) and agitated at 800 RPM for 5 min at room temp. Following centrifugation to remove the flow-through, peptide-bound Oligo R3 beads were washed twice: 0.1% formic acid was added and agitated at 800 RPM for 2 min, followed by centrifugation. Desalted peptides were eluted in 0.1% formic acid/30% acetonitrile and lyophilized in a speed-vacuum (Heto Cooling System). Dried peptides were stored at 4 °C until required.

### 2.6 Liquid chromatography coupled tandem mass spectrometry

Mass Spectrometry was performed at the Biological Mass Spectrometry Core Facility, the University of Manchester, according to previously published protocols (Eckersley et al., 2020). Digested samples were analyzed by LC-MS/MS using an UltiMate^®^ 3000 Rapid Separation LC (RSLC, Dionex Corporation, Sunnyvale, CA) coupled to a Q Exactive HF (ThermoFisher Scientific, Waltham, MA) mass spectrometer. Peptides were selected for fragmentation automatically by data dependent analysis. All MS/MS ions were searched using the Mascot engine (Matrix Science, London, UK; version 2.5.1). MS/MS ions were searched against the UniprotKb/SwissProt_2018_01 database (selected for *Homo sapiens*, with 161629 entries) assuming the digestion enzyme trypsin. Oxidation of methionine and proline (+16 Da) was specified in Mascot as a variable modification. The change of cysteine to a carbamidomethyl group (+57 Da) was selected as a fixed modification. Scaffold (Scaffold 5), Proteome Software Inc., Portland, OR) was used to filter for high confidence peptide and protein identifications. Peptide identifications were accepted if they could be established at greater than 90% probability by the Peptide Prophet algorithm as previously described (Eckersley et al., 2020) with Scaffold delta-mass correction. Protein identifications were accepted if they could be established at greater than 99% probability and contained at least 2 identified peptides. Protein probabilities were assigned by the Protein Prophet algorithm (Nesvizhskii et al., 2003). To make data comparison with MSP, Scaffold results were exported to MS Excel.

### 2.7 Peptide location fingerprinting

The Manchester Peptide Location Finger printer (MPLF) is a publicly available webtool (Ozols et al., 2021b) for analyzing LC-MS/MS data (www.manchesterproteome.manchester.ac.uk/#/MPLF). Following LC-MS/MS, Scaffold peptide lists were imported into the MPLF webtool. Within the tool, peptide spectrum matches (PSM) were mapped to and automatically summed within 50 amino acid steps along protein sequences, normalized against individual protein total spectrum count and the mean calculated per group (UV-irradiated versus non-irradiated). Peptides spanning two adjacent 50 amino acid steps were included in both steps. Mean PSM counts from each 50 amino acid step of a protein in one group (non-irradiated) were subtracted from the PSM counts of the corresponding 50 amino acid step of the same protein in the second group (UV-irradiated) and divided by 50 amino acids to identify regional differences in peptide yield. To identify significant changes to 50 amino acid steps between UV-irradiated and non-irradiated groups, a Bonferroni-corrected, repeated measures paired ANOVA test was used.

### 2.8 Protein interaction and pathway analysis

For the analysis of the complex protein mixture data, Uniprot identifiers corresponding to the 25 proteins with significant differences in local PSM counts following UVR exposure were submitted to the STRING database v11.5 (Szklarczyk et al., 2023) and searched against the *Homo sapiens* database. Local Network Cluster analysis was used with a false discovery rate cut-off of <0.001 to highlight related groups of UV susceptible proteins that demonstrated significant interaction, and Gene Ontology (GO) analysis was used with a false discovery rate cut-off of <0.01 to highlight enriched biological pathways involving UVR-susceptible proteins.

## 3 Results

### 3.1 Mature collagen I is resistant to UVR

**FIGURE 1:**
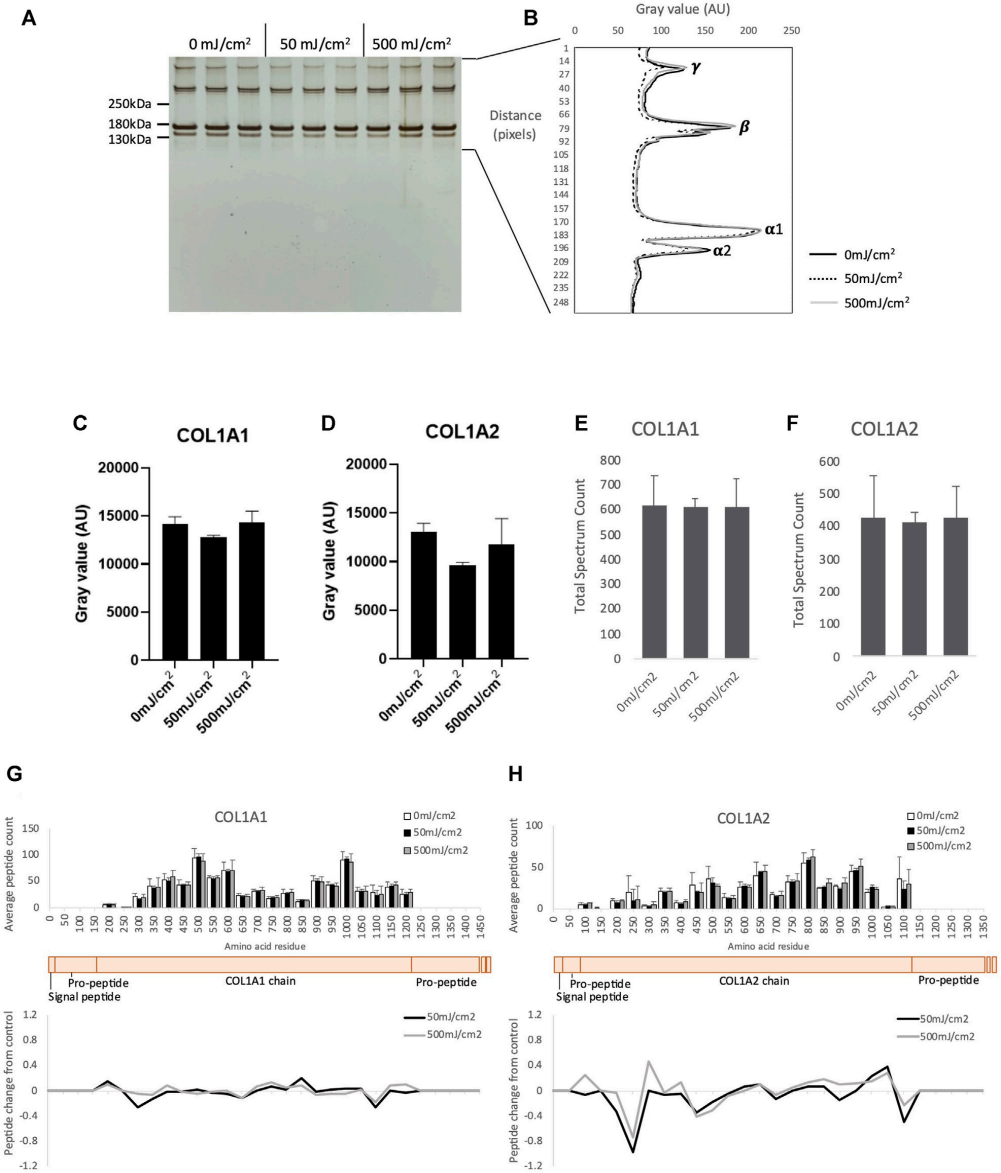
SDS-PAGE of purified native human collagen I irradiated with UV at 50- and 500 mJ/cm^2^ showing γ, β and α collagen (*n* = 3 per group) **(A)**. Electrophoretic mobility of UV irradiated γ, β and α collagen quantified by ImageJ **(B)**. Band intensity of COL1A1 **(C)** and COL1A2 **(D)** following UVR as measured by ImageJ. Peptide abundance (total spectrum count) of COL1A1 **(E)** and COL1A2 **(F)** irradiated with UV, assessed by LC-MS/MS. Peptide location fingerprint of COL1A1 **(G)** and COL1A2 **(H)** following UV at 50- and 500 mJ/cm^2^ showing average peptide count (bar chart) and relative change in peptide abundance from control (line graph) in each 50 amino acid step along the protein. Schematic of COL1A1 and COL1A2 (peach) with corresponding protein domains (modified from Uniprot). AU, arbitrary units; Error bars in all graphs indicate standard deviation.

Having previously shown that purified, mature collagen I appears resistant to UVR (both broadband UVB and SSR) by gel electrophoresis (Sherratt et al., 2010) we aimed to confirm this apparent UVR-resistance using the more sensitive PLF technique. Collagen I extracted from placenta was irradiated with broadband UVB, at 1X MED (50 mJ/cm^2^) and 10X MED (500 mJ/cm^2^). As previously reported (Sherratt et al., 2010), native collagen I was resistant to UVR (Figures 1A–H): following irradiation the electrophoretic mobility of alpha chains and higher order assemblies (β and γ) (Figure 1B) and the intensity of COL1A1 (α1) and COL1A2 (α2) bands (Figures 1C, D), was unaltered. PLF was then employed to determine if UVR exposure induced structural changes in collagen I which were not detectable by SDS-PAGE. UVR exposure at 50- and 500 mJ/cm^2^ did not significantly alter either the abundance of COL1A1 or COL1A2, as judged by total spectrum count (Figures 1E, F), or the structure of COL1A1 and COL1A2, as assessed by PLF (Figures 1G, H; Supplementary Table S1). As predicted by amino acid composition (UV chromophore and ROS-susceptible amino acid content of COL1A1 and COL1A2 is 1.4% and 2.2%, respectively) and determined previously by experiment, mature (and therefore processed) collagen I appears resistant to a UVR source comprising predominantly UVA and UVB wavelengths.

### 3.2 Tissue fibronectin is susceptible to UVR

**FIGURE 2:**
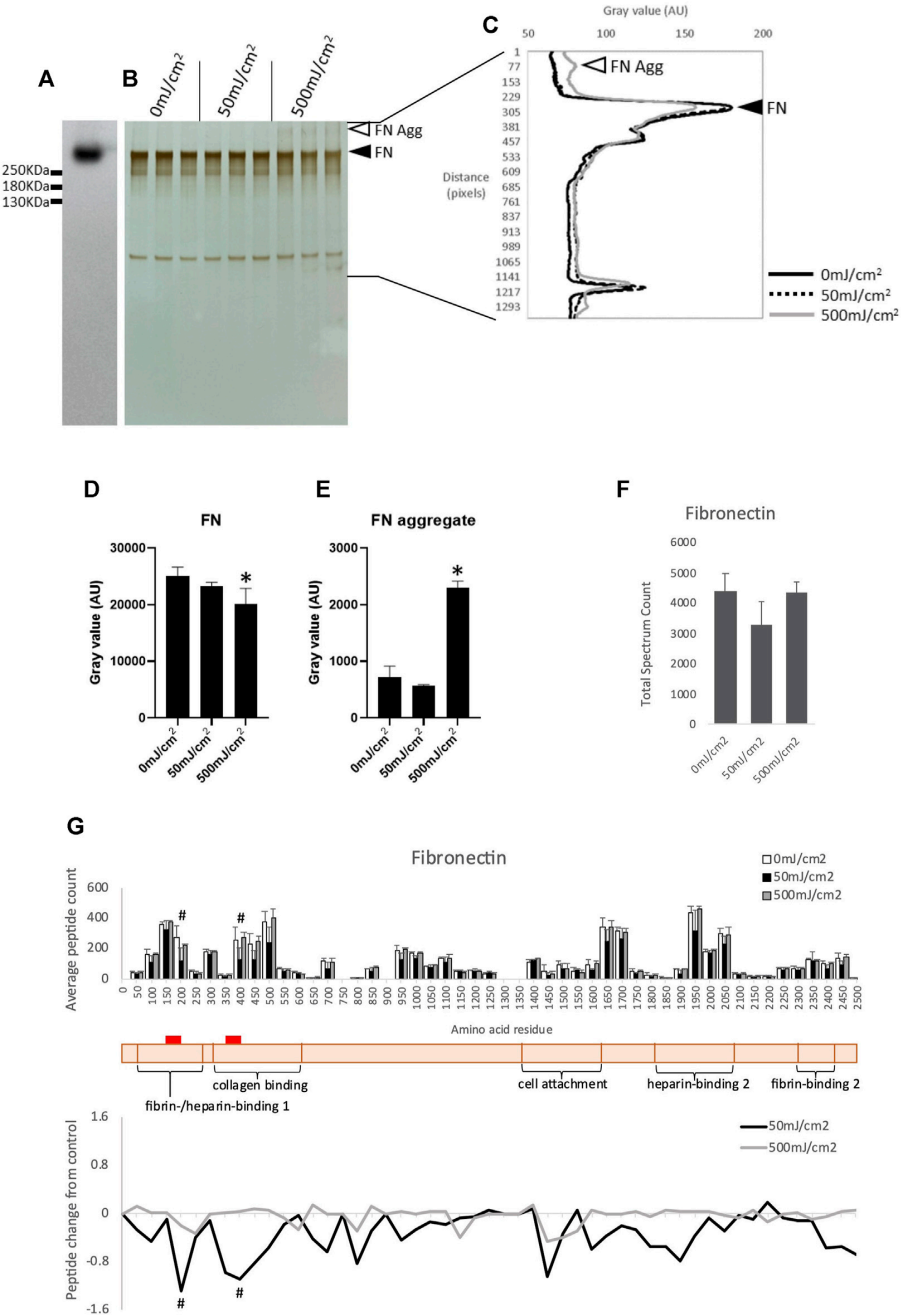
Western blot of purified human tissue fibronectin **(A)**. SDS-PAGE of human tissue fibronectin irradiated with 50- and 500 mJ/cm^2^ UV, showing fibronectin band (adjacent to filled arrowhead) and aggregated fibronectin (adjacent to unfilled arrowhead) **(B)**. Electrophoretic mobility of UV irradiated fibronectin quantified by ImageJ **(C)**. Intensity of tissue fibronectin band (adjacent to filled arrow head), measured by ImageJ, showing a significant reduction in intensity in fibronectin irradiated with 500 mJ/cm^2^ **(D)**. Intensity of fibronectin aggregate band (adjacent to unfilled arrow head) showing a significant increase in intensity in fibronectin irradiated with 500 mJ/cm^2^ **(E)**. Peptide abundance (total spectrum count) of tissue fibronectin irradiated with UV, assessed by LC-MS/MS **(F)**. Peptide location fingerprint of tissue fibronectin following UV at 50- and 500 mJ/cm^2^ showing average peptide count (bar chart) and relative change in peptide abundance from control (line graph) in each 50 amino acid step along the protein **(G)**. Schematic of fibronectin (peach) with corresponding protein domains (modified from Uniprot). Red bar indicates UV-susceptible domains (150–200 and 350–400). *p < 0.05 (0 mJ/cm^2^ vs. 500 mJ/cm^2^), one-way ANOVA with Dunnett’s multiple comparison *post hoc* test; #*p* < 0.05 (0 mJ/cm^2^ vs. 50 mJ/cm^2^), Bonferroni-corrected, repeated measures paired ANOVA. Error bars in all graphs indicate standard deviation. AU, arbitrary units.

Exposure to UVR induces aggregation of plasma fibronectin (FN) in a dose dependent manner (Sherratt et al., 2010). Although plasma FN is used in many studies of the human ECM it is structurally distinct from tissue FN (Dalton and Lemmon, 2021), which is the main FN isoform in skin. In contrast to collagen I, both isoforms of FN are rich in UV chromophore and ROS-susceptible amino acid residues (11.6%) and hence are predicted to be susceptible to UVR and/or photo-dynamically produced ROS. Therefore, tissue FN was used in this investigation. As other low molecular weight species were observed in the commercially available Fn preparation by SDS-PAGE the presence of intact FN (single band of >250 KDa) was confirmed by Western blotting (Figures 2A, B). Compared to unirradiated controls, exposure to a UVR dose of 500 mJ/cm^2^ induced significant aggregation of FN (*p* < 0.05, Figures 2C, E) and reduction in FN band intensity (*p* < 0.05, Figures 2C, D). These observations demonstrate that both plasma (Sherratt et al., 2010) and tissue FN experience similar remodeling as characterized by SDS-PAGE although higher UVR doses are required to induce aggregation of tissue FN.

Proteomic analysis demonstrated that UVR exposure at 50- and 500 mJ/cm^2^ did not significantly alter the abundance of FN as judged by total spectrum count for the whole proteins (Figure 2F). However, PLF identified a significant local reduction in the number of trypsin-liberated peptides in 50 aa segments located within the fibrin-/heparin-binding domain-1, and the collagen-binding domain of tissue FN irradiated with 50 mJ/cm^2^ (Figure 2G; Supplementary Table S2). Therefore, in agreement with their respective chromophore content, and when analyzed by the sensitive PLF MS technique, type-I collagen appears resistant to and tissue FN susceptible to broadband UVR. Therefore, PLF analysis has the potential to screen complex protein mixtures for UV-susceptible targets and we next used cultured dermal fibroblasts to synthesize a complex matrix to act as a UVR target.

### 3.3 Cultured human dermal fibroblasts deposit an ECM which is comparable to the human dermis matrisome

The dermis is a complex environment. Using human skin biopsies, we have previously identified more than 900 dermal proteins which are sufficiently abundant to be detected by LC-MS/MS (Ozols et al., 2021b). It is unlikely, therefore, that the only dermal proteins which are susceptible to UVR are those already identified by targeted experiments on purified protein suspensions (i.e., fibrillin microfibrils, fibronectin [now both plasma and tissue] (Sherratt et al., 2010). In order to identify other potential UVR-susceptible ECM targets PLF was used to screen a dermal fibroblast-derived ECM-enriched proteome.

**FIGURE 3:**
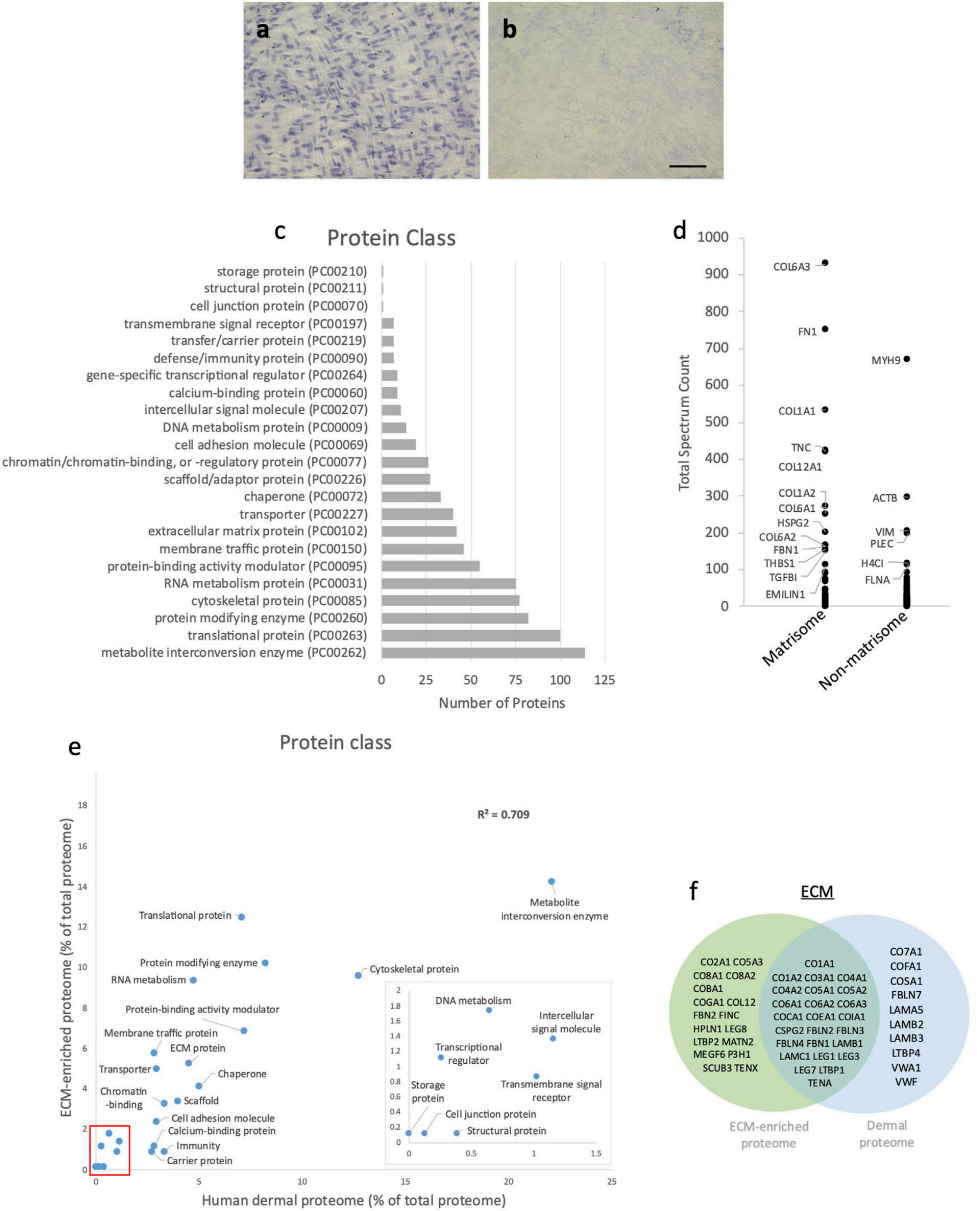
Characterisation of ECM-enriched proteome. Human dermal fibroblast cell culture before **(A)** and after **(B)** cell removal; nuclei stained with haematoxylin (scale bar = 100 μm and applies to all images). Classification of the ECM-enriched proteome by PantherDB **(C)**; of the 977 proteins identified following LC-MS/MS, PantherDB classified 803 proteins into 23 protein classes. Abundance (mean total spectrum count) of matrisome and non-matrisome proteins in ECM-enriched proteome **(D)**; labelled proteins have a mean total spectrum count >100). Correlation of the percentage of proteins present within each protein class in ECM-enriched proteome and human dermal proteome (*R* ^2^ = 0.709), classified by PantherDB (red box indicates area of magnified insert graph) **(E)**. Venn diagram showing shared and unique matrix proteins within the ECM-enriched proteome and human dermal proteome **(F)**.

Cells were removed by an established lysis protocol (Franco-Barraza et al., 2016). Following lysis and washing, no haematoxylin-positive cell nuclei were visible (Figures 3A, B). LC-MS/MS followed by protein identification of the cell-depleted cultures by Mascot identified a total of 977 individual proteins (Supplementary Table S3) classified by PantherDB (803 classified and 174 unclassified) into 23 protein classes (Figure 3C). The major protein classes (>80 proteins) were metabolite interconversion enzymes (114), translational proteins (100) and protein modifying enzymes (82). Although the ECM protein class contained only 42 discrete protein hits, the most abundant individual proteins (judged by total spectrum count) including COL6A3, FN and COL1A1 were ECM components (Figure 3D). COL6A3 and FN have previously been identified as abundant ECM components in this model system (Ghetti et al., 2018) and in skin (Li et al., 2022). As many intracellular proteins were present in relatively low-abundance this cell-depleted proteome can be considered to be ECM-enriched.

To determine how closely the ECM-enriched proteome models the *in vivo* dermal matrix the ECM-enriched proteome was compared with a mass spectrometry derived human dermal proteome (Ozols et al., 2021b). Following classification by PantherDB, the human dermal proteome contained 779 proteins which categorized into 22 protein classes, which were the same as 22 out of the 23 protein classes (minus the “storage proteins” class) represented in the ECM-enriched proteome (Figure 3E). The proportion of proteins within each protein class in the human dermal proteome showed a strong correlation with the ECM-enriched proteome (*R* ^2^ = 0.709), particularly for ECM proteins, which comprised 4.5% of the human dermal proteome and 5.2% of the ECM-enriched proteome. Although some proteins were unique to the ECM-enriched proteome, including fibronectin and fibrillin-2 (Figure 3F) this analysis indicates that cultured dermal fibroblasts synthesize many dermal ECM proteins (i.e., fibrillar collagens (I, III and V), collagen VI, tenascin-C, versican and fibrillin-1) with sufficient abundance to serve as a dermal model.

### 3.4 PLF identifies a sub-population of novel UVR-susceptible ECM proteins

**FIGURE 4:**
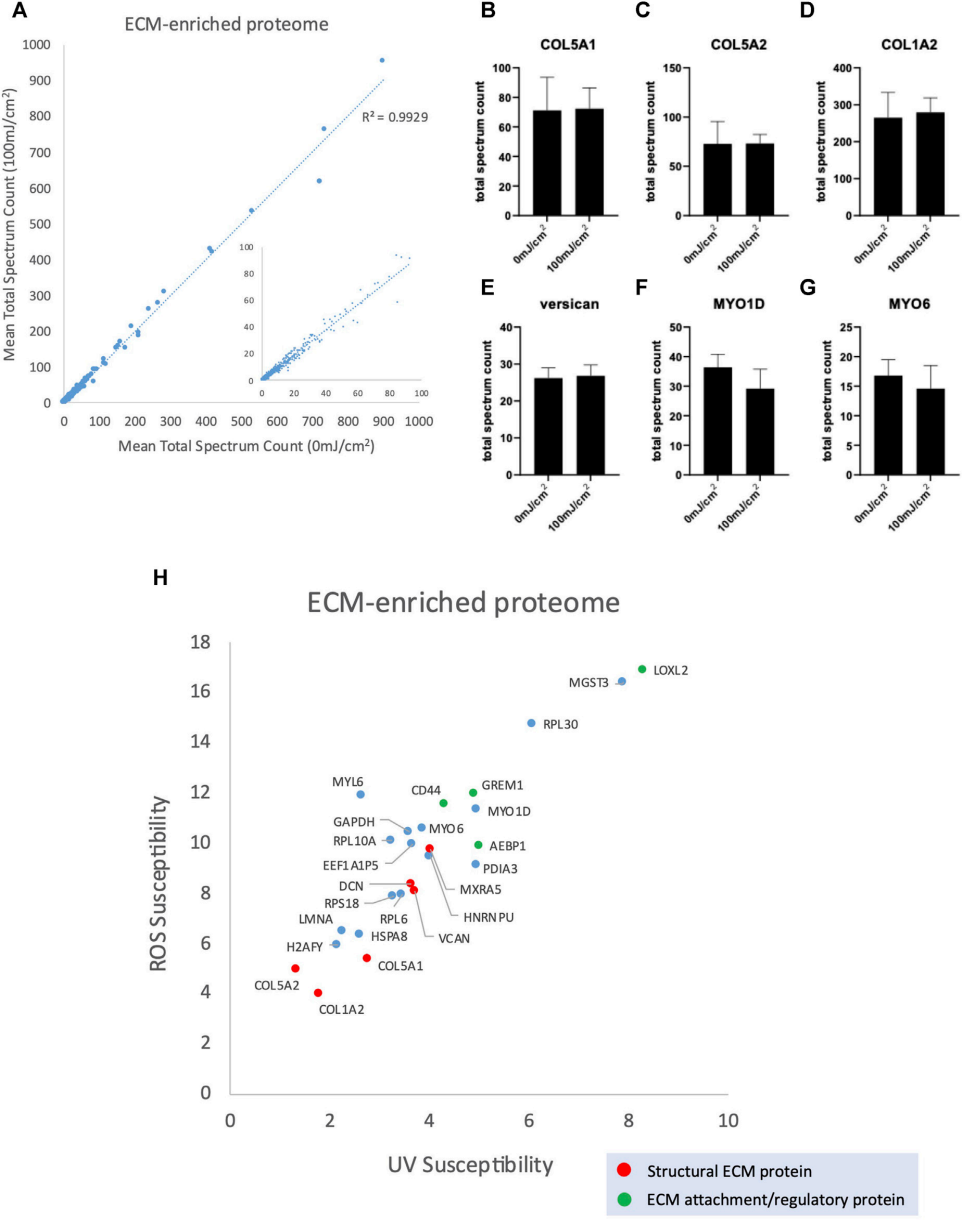
Peptide abundance (mean total spectrum count) of ECM-enriched proteome irradiated with 100 mJ/cm^2^ compared with 0 mJ/cm^2^ (*R* ^2^ = 0.9929) **(A)**, inset scatter graph shows proteins with a mean total spectrum count <100. Peptide abundance (mean total spectrum count) of COL5A1 **(B)**, COL5A2 **(C)**, COL1A2 **(D)**, versican **(E)**, MYO1D **(F)** and MYO6 **(G)**, showing no significant difference in abundance between UV-irradiated (100 mJ/cm^2^) and non-irradiated control. Scatter graph showing UV/ROS susceptibility values for the 25 proteins in ECM-enriched proteome that are structurally altered in response to 100 mJ/cm^2^ UVR **(H)**.

The ECM-enriched proteome was irradiated with 100 mJ/cm^2^, a UV dose which mimics multiple UVR exposures. There was a strong correlation between protein abundance in the UV irradiated ECM-enriched proteome and non-UV irradiated (Figure 4A; *R* ^2^ = 0.9929) and no significant differences between the abundance of individual ECM proteins (4b-g), indicating that peptide spectrum counts, and therefore relative protein abundance, are not affected by UV exposure (Supplementary Table S3). Therefore, conventional LC-MS/MS analysis, which characterizes proteomes with regards to protein identity and relative abundance, is not well-suited to identifying targets of UVR-induced damage. However, PLF analysis identified 25 proteins displaying significant structural changes following UVR exposure, of which 6 were structural ECM proteins (red) and 4 are ECM attachment/regulatory proteins (green) (Figure 4H; Supplementary Table S4). We have previously shown that amino acid composition (with regards to UVR chromophore and/or oxidizable residues) is predictive of relative susceptibility to UVR and/or photodynamic ROS (Sherratt et al., 2010) and have confirmed, in this study, that mature collagen I extracted from tissues is UVR-resistant. However, following UVR-irradiation, the constituent alpha chains of fibrillar collagen I and V (COL1A2, COL5A1, COL5A2) were identified as UVR-susceptible. Following initial synthesis, fibrillar collagen undergoes extensive post-translational modification and assembly. Therefore, PLF has the potential to distinguish between damage to immature and mature forms of ECM components.

### 3.5 UVR targets proteins involved in ECM collagen fibril organization

**FIGURE 5:**
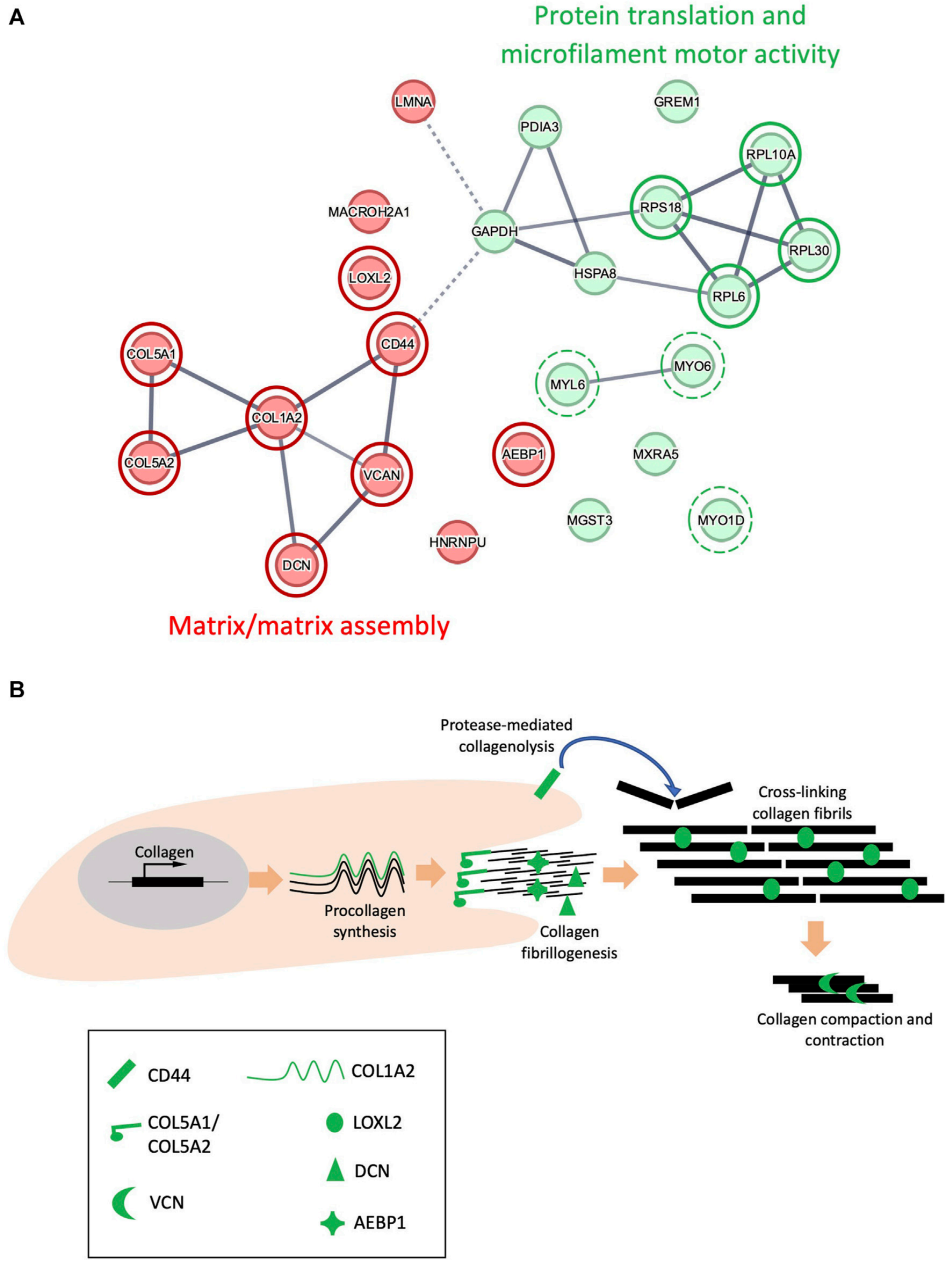
STRING interaction networks of UVR-modified proteins. Interactions between 24 proteins in the ECM-enriched proteome that are structurally altered following exposure to 100 mJ/cm^2^ UVR, evaluated by STRING **(A)**. UV-susceptible proteins divided into 2 groups (red/green) using k-means clustering, based upon similarity in co-expression, text mining and protein homology. Circles with a red border highlight proteins involved in matrix/matrix assembly. Circles with a green border highlight proteins involved in protein translation, and circles with a broken green border highlight proteins involved in microfilament motor activity. Line intensity is proportional to the strength of interaction between 2 proteins (FDR <0.001). Schematic indicating the potential role of COL1A2, COL5A1, COL5A2, LOXL2, versican (VCN), decorin (DCN), CD44 and AEBP1 in collagen fibrillogenesis **(B)**.

To further understand the relationship between UV susceptible proteins in the ECM-enriched proteome, protein interactions were assessed using the search tool for the retrieval of interacting genes/proteins (STRING) database. STRING recognized 24 out of 25 proteins inputted (EEF1A1P5 was not recognized) which were divided into 2 main groups using k-means clustering (Figure 5A): (I) Matrix/matrix assembly, (II) Protein translation and microfilament motor activity. Local network clustering (STRING), Gene ontology (GO) analysis and a literature search indicated that 8 of the 11 proteins identified in group 1 were involved in collagen fibrillogenesis and ECM organization: COL1A2, COL5A1, COL5A2, decorin, versican, CD44, LOXL2 and AEBP1. The reported roles of these proteins collagen fibrillogenesis are depicted in Figure 5B.

**FIGURE 6:**
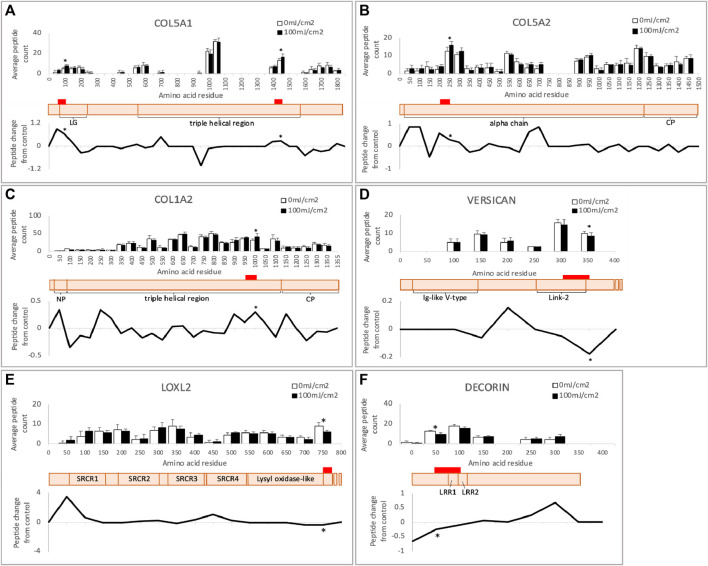
Peptide location fingerprint of COL5A1 **(A)**, COL5A2 **(B)**, COL1A2 **(C)**, versican **(D)**, LOXL2 **(E)** and decorin **(F)** in ECM-enriched proteome following UV at 100 mJ/cm^2^, showing average peptide count (bar chart) and relative change in peptide abundance from control (line graph) in each 50 amino acid step along the protein. Schematic of susceptible proteins (peach) with corresponding domains (modified from Uniprot). Red bars indicate UV-susceptible domains. *p < 0.05, Bonferroni-corrected, repeated measures paired ANOVA. Error bars in all graphs indicate standard deviation. Graph for versican is truncated at 400 aa for clarity. LG, laminin G-like domain; NP, N-terminal propeptide; CP, C-terminal propeptide; LRR, leucine-rich repeat; SRCR, scavenger receptor cysteine-rich.

In addition to identifying susceptible proteins and hence the potential impact on protein-protein interactions, PLF can highlight regions within proteins which exhibit UVR-mediated differential susceptibility to tryptic cleavage and hence structural variations. Two such regions were identified in COL5A1 at residues 50-100 (within the laminin G-like domain) and 1400-1450 (triple helix) (Figure 6A). The protein region altered by UVR in COL5A2 (200–250) is within the main triple helix towards the C-terminus (Figure 6B). Although mature collagen I alpha chains appear to be resistant to relatively high doses of broadband UVB (Sherratt et al., 2010 and this study), the newly synthesized alpha 2 chain of collagen I contains a region within the triple helix which is structurally compromised following UVR exposure (Figure 6C). In versican, significantly altered local peptide abundance following UVR is found spanning the Link-2 module of the G1 domain, a region of the protein that interacts with hyaluronan, and the glycosaminoglycan (GAG) attachment domain (300–350) (Figure 6D). In LOXL2, UV-induced changes occur at the protein’s c-terminus (750–774) adjacent to the catalytic domain (Figure 6E) and, in decorin, UV-induced changes span leucine-rich repeat domains 1 and 2 (50–100) (Figure 6F).

### 3.6 Intracellular proteins involved in protein synthesis are also targets of broadband UVB radiation

Local network clustering (STRING), gene ontology (GO) analysis and literature searches indicated that 4 of the 13 proteins identified in the ‘protein translation and microfilament motor activity’ group were components of the ribosome: RPS18, RPL30, RPL10A, RPL6; in addition to 3 motor proteins: MYO1D, MYO6, MYL6 (Figure 5A). However, these results may not be representative of the response of intracellular proteins to UV, as cells were removed from the matrix prior to UV irradiation, thus exposing intracellular proteins to an extracellular environment.

## 4 Discussion

This study aimed to confirm, using alternative characterization techniques, the apparent resistance to UVR-induced damage of collagen I and susceptibility of FN to UVR and to screen a complex dermal model proteome for UVR susceptible targets.

Using techniques with enhanced sensitivity (silver stain, PLF) this study confirmed earlier findings (Sherratt et al., 2010) showing that mature collagen I is resistant to physiological and supra-physiological doses of UVR. However, screening of cell culture-derived proteomes demonstrated that newly synthesized collagen I was structurally altered in response to low-dose UVR. Mature collagen I extracted from tissues has undergone fibrillogensis prior to disaggregation and solubilization in acidic conditions. However, the detection of the N- and C-pro-peptide regions of COL1A1 and COL1A2 in the cell culture-derived proteome demonstrates that at least some of the collagen is present as the soluble, non-cross-linked precursor pro-collagen I. We have previously shown using an early form of PLF that the tryptic cleavage pattern of cell culture- and tissue-derived fibrillin microfibrils differs (Eckersley et al., 2018). Therefore, the relative susceptibility of newly synthesized collagen (and other ECM components) to UV and/or ROS may have implications for processes in which collagen synthesis/remodeling occurs including normal proteostasis and wound healing. *In vivo* studies show that collagen synthesis is modulated by ageing but not by UVB (Autio et al., 1994), and *in vitro* studies show that collagen-I fibrillogenesis *in vitro* is relatively resistant to high UVR doses and solar-simulated radiation (Menter et al., 2001).

PLF of FN exposed to 50 mJ/cm^2^ UVR highlighted two variable regions towards the N-terminus corresponding to the FN type-I^4-5^ domain and the FN type-II^1-2^ domain, which bind fibrin/heparin and collagen, respectively (Dalton and Lemmon, 2021). The type-I^4-5^ domain is essential for fibril assembly (Schwarzbauer, 1991), and critical for the binding of hyaluronan which facilitates attachment of epithelial cells (Nakamura et al., 1994). The FN type-II^1-2^ domain is necessary for normal ECM organization, as blocking this domain results in reduced collagen deposition by human dermal fibroblasts *in vitro* (McDonald et al., 1982). Aggregation of UV irradiated fibronectin is likely to be due to the action of photo-dynamically produced ROS. We have previously shown that UVR mediated fibronectin aggregation is abrogated in depleted O_2_ conditions and enhanced in the presence of D_2_O (Hibbert et al., 2019). Structural changes were observed in purified tissue FN but not FN in ECM-enriched proteome. This may be explained by the relative stability of FN when assembled into multimeric insoluble fibrils (Sottile and Hocking, 2002; Wierzbicka-Patynowski and Schwarzbauer, 2003) which are functionally different from soluble fibronectin (Morla et al., 1994). Whilst beyond the scope of this study, it is clear that changes in fibronectin structure (by for example, physiological loading) can expose hydrophobic regions which drive fibronectin self-association (Gee et al., 2008) and that cell interactions with FN are influenced by the proximity of the RGD and synergy sites (Craig et al., 2008). As a consequence UVR-induced changes in FN structure have the potential to impact on normal tissue function.

Irradiation of tissue FN with 500 mJ/cm^2^ UVR reduced the intensity of the FN protein band and induced aggregation (Figures 2A–C). However PLF did not detect statistically significant changes following high-UVR exposure (Figure 2G). We have previously shown that PLF is well-suited to detecting changes in peptide yield and hence fingerprint where control proteins (non-UV exposed or young) exhibit a conserved fingerprint and any induced changes (due to UVR exposure or ageing) are also conserved (Eckersley et al., 2022). However, where proteins are subject to extensive and potentially stochastic changes in structure and hence tryptic cleavage (as indicated by the aggregation and smearing observed in Figures 2A–C) the PLF technique may struggle to identify significant changes in peptide yield. It is important therefore to use complimentary techniques to detect structural changes in proteins.

The final aim of the study was to determine if PLF could identify structurally compromised proteins following UVR exposure in a complex proteome. Unlike histology or proteomic techniques that measure protein abundance, PLF can identify specific protein regions that are susceptible to UV damage. In this study we used cells from a single human donor as a source of ECM proteins. We have previously shown using PLF that, for many ECM proteins, their peptide fingerprint is remarkably well conserved between cells and tissues from different individuals and even between different species (Eckersley et al., 2020, 2022, 2023). However, it is also the case that, due to the statistical nature of PLF analyses, UVR-induced changes in lower abundance proteins may be missed in our current analysis. Therefore PLF analysis of irradiated proteomes produced by cells from alternative donors, with different protein abundance profiles, may identify additional UVR-sensitive proteins.

The relative proportion of UV- and ROS-susceptible amino acids is highly variable within the 25 UV-susceptible proteins identified here, and ranges from proteins with high UV/ROS-susceptibility, such as LOXL2 and MGST3, to proteins with low UV/ROS susceptibility, such as COL1A2 and COL5A2. This suggests that although the proportion of UV/ROS-sensitive amino acids can predict the relative UV susceptibility of some proteins, this explanation does not apply in all cases, indicating that additional mechanisms are responsible for UV/ROS-induced protein damage.

To uncover possible relationships between UV-susceptible proteins, the cohort of 25 PLF-identified UVR susceptible proteins was analyzed for STRING interactions, gene ontology and by reference to the literature. This analysis revealed a prominent cluster of UVR-susceptible proteins involved in collagen structure and fibrillogenesis (Figure 5: “Matrix/Matrix assembly”). Collagen V is composed of 3 distinct alpha chains, which are found as heterotrimeric (α1(V)_2_α2(V), α1(V)α2(V)α3(V) and homotrimeric (α1(V)_3_) fibrils in skin. Heterotrimeric collagen V fibrils regulate the diameter of fibrillar collagen I fibrils in skin (Wenstrup et al., 2004) and mutations in *COL5A1* and *COL5A2* can result in classic Ehlers-Danlos syndrome, characterized by fragile skin, hyperextensible skin and impaired wound healing. Collagen V may also be sensitive to age-related changes, as the structure of COL5A1 is altered in aged tendon (Ozols et al., 2021b). In the present study two regions of COL5A1 (triple helical and laminin G-like domains) and one region of COL5A2 (triple helical) are structurally altered in response to UVR. As previously discussed in relation to COL1A2, newly synthesized triple helical regions of COL5A1 and COL5A2 in an ECM-enriched proteome may show increased susceptibility to UVR compared with the mature, native protein isolated from tissue. The laminin G-like domain of COL5A1 is located within the N-terminal propeptide (Paladin et al., 2015), a region which is retained by the mature protein, and which interacts with and regulates the diameter of collagen-I (Symoens et al., 2011). Because the amino terminal domain of collagen-V regulates collagen-I fibril diameter, UV damage to the amino terminal domain of COL5A1 may affect normal collagen fibrillogenesis.

PLF analysis of the irradiated ECM-enriched proteome identified other potential UVR targets including lysyl oxidase-like 2 protein (LOXL2); an amine oxidase involved in the formation of lysine-derived cross-links in both collagen and elastin (Kim et al., 2011; Schmelzer et al., 2019). Adipocyte enhancer binding protein 1 (AEBP1) also known as aortic carboxypeptidase-like protein - ACLP) is the larger isoform encoding a secreted protein that binds to collagen and regulates fibrillogenesis, (Majdalawieh et al., 2020). Small leucine rich proteoglycans (SLRPs) also play a key role in collagen fibrillogenesis. Decorin is an SLRP which regulates collagen I fibril formation (Rühland et al., 2007) in skin and tendon. Its targeted disruption in mice results in abnormal collagen architecture leading to fragile skin (Danielson et al., 1997) and in humans decorin staining is greatly diminished in photodamaged skin (Bernstein et al., 1995). Another SLRP: versican, is a large proteoglycan which modulates collagen compaction and contraction during *in vitro* fibrillogenesis (Chen et al., 2020) and is localized predominantly to the reticular dermis (du Cros et al., 1995). Finally, CD44 is a transmembrane cell adhesion receptor that binds to extracellular hyaluronan. CD44 knockout mice display enhanced collagen accumulation in skin due to a decrease in protease-mediated collagenolysis (Govindaraju et al., 2019), suggesting that CD44 regulates the abundance of dermal collagen post-wounding. The cytoplasmic domain of CD44, shown to be UV susceptible in our study, is also important for chondrocyte pericellular matrix assembly (Jiang et al., 2002).

In addition to ECM proteins, the ECM-enriched proteome contained intracellular proteins such as ribosomes and the cytoskeletal proteins actin and myosin. UVR induces significant changes to proteins in the 60S subunit (RPL6, RPL10A, RPL30) and the 40S subunit (RPS18). Ribosomes are composed of a large 60S subunit and small 40S subunit, each containing many individual ribosomal proteins that collectively synthesize proteins (Wilson and Doudna Cate, 2012). A previous study has shown that ribosomal subunits RPS26 and RPL10, damaged by hydrogen peroxide, are selectively removed from the ribosome and replaced with non-damaged proteins, resulting in ribosome repair (Yang et al., 2023). Thus, ribosomal proteins susceptible to damage by hydrogen peroxide (a ROS), may also be susceptible to direct UV/UV-induced ROS damage. However, the study by Yang does not evaluate the effects of ROS-mediated ribosomal damage on protein translation. In the present study, UV alters the structure of 3 motor proteins, MYO1D, MYO6 and MYL6. Myosin motor proteins use ATP hydrolysis to translocate along actin filaments and are involved in many cell processes including muscle contraction, cell migration, endocytosis, and vesicle trafficking (Sweeney et al., 2020). Thus, low-dose UV may alter the function of intracellular proteins involved in protein translation and cell movement. However, exposure of intracellular proteins to an extracellular environment prior to UV irradiation, due to cell removal, may have altered their susceptibility to UV, therefore caution should be used when interpreting this observation.

UV-induced oxidation is a likely mechanism responsible for the structural changes to dermal ECM proteins reported in this study. We have previously shown that UV-induced structural changes in dermal ECM components, such as fibrillin microfibrils and fibronectin, are mediated in part by photo-oxidation (Hibbert et al., 2019). Topical antioxidants may therefore have the potential to inhibit protein oxidation in UV-irradiated skin. Using the SKH-1 hairless mouse model, Sirerol et al. (2015) showed that an analog of resveratrol, a polyphenol found in grapes, reduced the level of protein carbonyls in skin following UV irradiation. Furthermore, synthetic antioxidants (nitroxides) inhibit UV-A induced damage and carbonyl accumulation in calf dermal collagen III *in vitro* (Venditti et al., 2008). Sunscreens, which protect skin by blocking absorption of UV radiation and preventing UV-mediated photooxidation of dermal proteins may also play a role in reducing ECM damage. Sunscreens have been shown to reduce protein carbonylation in *ex vivo* human skin explants irradiated with high energy visible (HEV) light (Francois-Newton et al., 2022). *In vitro* evidence shows that flat spectrum sunscreen prevents damage to purified fibronectin and fibrillin microfibrils, induced by solar-simulated radiation (Hibbert et al., 2017).

## 5 Conclusion

Using more sensitive techniques (silver staining, LC-MS/MS followed by PLF) this study confirms that human collagen I is resistant to UV doses up to and including 500 mJ/cm^2^. Human tissue FN displays structural changes in response to UVR at 50 mJ/cm^2^, within its fibrin/heparin-binding and collagen-binding domains. In addition, tissue fibronectin shows aggregation following UVR at 500 mJ/cm^2^. Acute, low-dose UV targets a small group (25) of proteins within a larger ECM-enriched proteome. Cluster analysis of the UV-susceptible protein group, using STRING combined with gene ontology analysis, highlights a cluster of proteins involved in collagen fibrillogenesis. Analysis of protein structure reveals that the protein domains selectively targeted by UV are involved in ECM binding. In conclusion, this study identifies extracellular matrix proteins that are structurally altered in response to low-dose UVR and establishes a role for PLF in screening biological systems for UVR and ROS-mediated damage.

## Data Availability

The mass spectrometry datasets for this study can be found in the jPOST online repository: https://repository.jpostdb.org/preview/75786948765e9eef1af246, access code (6602).
